# The impact of isolated maternal hypothyroxinemia during the first and second trimester of gestation on pregnancy outcomes: an intervention and prospective cohort study in China

**DOI:** 10.1007/s40618-018-0960-7

**Published:** 2018-10-17

**Authors:** X. Gong, A. Liu, Y. Li, H. Sun, Y. Li, C. Li, X. Yu, C. Fan, Z. Shan, W. Teng

**Affiliations:** 1 Department of Endocrinology and Metabolism, Institute of Endocrinology, Liaoning Provincial Key Laboratory of Endocrine Diseases, The First Affiliated Hospital of China Medical University, Shenyang, 110001 People’s Republic of China; 2 Department of Endocrinology and Metabolism, Anshan Central Hospital, Anshan, 114001 People’s Republic of China; 3 Department of Rheumatism and Hematology, First Hospital of Dandong, Dandong, 118000 People’s Republic of China

**Keywords:** First and second trimester, Levothyroxine (L-T4), Isolated maternal hypothyroxinemia (IMH), Pregnancy outcomes

## Abstract

**Objectives:**

To explore the effect of isolated maternal hypothyroxinemia (IMH) during the first and second trimester of gestation on pregnancy outcomes. To explore whether levothyroxine (L-T4) treatment of women who had IMH identified in the first trimester improves pregnancy outcomes.

**Methods:**

Women in the early pregnancy in the iodine-sufficient area (*n* = 3398) were recruited to this prospective cohort study (ChiCTR-TRC-12002326). Serum thyroid-stimulating hormone (TSH), free thyroxine (FT4), and thyroid peroxidase antibody (TPOAb) were detected. Women with IMH before 12 weeks chose to receive L-T4 or remain untreated. The L-T4 dose was adjusted to attain a normal FT4 and TSH level. Pregnancy outcomes were evaluated during follow-up.

**Results:**

IMH in the first trimester was not associated with increased risk of adverse pregnancy outcome compared with controls. The incidence of macrosomia (*p* = 0.022) and gestational hypertension (*p* = 0.018) was significantly higher in IMH identified in the second trimester of gestation compared with controls. IMH identified in the second trimester of gestation was a risk factor for macrosomia [adjusted odds ratio (aOR) 1.942, 95% CI 1.076–3.503, *p* = 0.027] and gestational hypertension (aOR 4.203, 95% CI 1.611–10.968, *p* < 0.01), when body mass index in the early pregnancy was < 25 kg/m^2^.

**Conclusions:**

IMH in the first trimester did not increase the risk of adverse outcomes irrespective of whether women received L-T4 treatment. However, IMH identified in the second trimester was associated with increased risk of adverse pregnancy outcome. The results suggest that thyroid function follow-up during the second trimester is necessary, even if thyroid function is normal during the first trimester.

**Electronic supplementary material:**

The online version of this article (10.1007/s40618-018-0960-7) contains supplementary material, which is available to authorized users.

## Introduction

Thyroid hormones are crucial for a normal pregnancy and fetal development. The physiological changes during pregnancy may lead to increased demand for thyroid hormones [1]. Non-optimal thyroid hormone availability during pregnancy is associated with adverse pregnancy outcomes, with increasing evidence showing that subclinical thyroid disease during pregnancy, particularly during early gestation, may threaten the health of both mother and their child, and cause cognitive deficits and psychological disorders in children in later life [1–3].

Isolated maternal hypothyroxinemia (IMH) is defined as a low maternal free thyroxine (FT4) concentration in conjunction with a normal maternal thyroid-stimulating hormone (TSH) concentration. IMH during the early pregnancy is associated with the increased risk of placental abruption, gestational diabetes, macrosomia, and preterm delivery [2, 4, 5]. IMH in the second trimester of gestation may also result in adverse pregnancy outcomes. The Amsterdam ABCD cohort study showed that women with an increase in TSH > 3.68 mIU/L or decrease in FT4 < 2.5th percentile (FT4 < 6.7 pmol/L) in the second trimester of gestation were at increased risk of breech delivery [6]. The Generation R study in the Netherlands found that decreased maternal serum FT4 in the first half of pregnancy caused a 2.5- and 3.9-fold increased risk in premature and very premature delivery, respectively [7]. However, Casey et al. [8] indicated that hypothyroxinemia in the first half of pregnancy had no effect on perinatal outcomes. A review by Korevaar et al. indicated that hypothyroxinemia may be associated with child neurocognitive development but not with adverse pregnancy outcomes [1].

Evidence about pregnancy outcomes following levothyroxine treatment of women with IMH is limited. Thus, the guidelines of the American Thyroid Association recommend that isolated hypothyroxinemia should not be routinely treated in pregnancy [9]. The European Thyroid Association guidelines recommend that levothyroxine therapy may be considered for IMH detected in the first trimester because of its association with neuropsychological impairment in children [10].

By evaluating the thyroid function of pregnant women, we explored the effect of IMH in the first and second trimester of gestation on pregnancy outcomes and investigated whether levothyroxine (L-T4) intervention beginning before 12 gestational weeks could reduce IMH-related complications.

## Materials and methods

### Subjects

This prospective cohort study was designed to investigate the associations between IMH and birth outcomes. Pregnant women participating in the Subclinical Hypothyroid in Early Pregnancy (SHEP) study were recruited. The SHEP study was started in June 2012 in Shenyang and Dalian cities of Liaoning Province in northeastern China, where iodine availability was adequate [11]. Women with a twin pregnancy, with a personal or family history of inherited disease (hypertension, cardiac disease, and diabetes), or with a history of other severe chronic diseases were excluded. Women who used medication for known thyroid disease and those with gestational week or time of blood sampling unknown were also excluded.

**Fig. 1:**
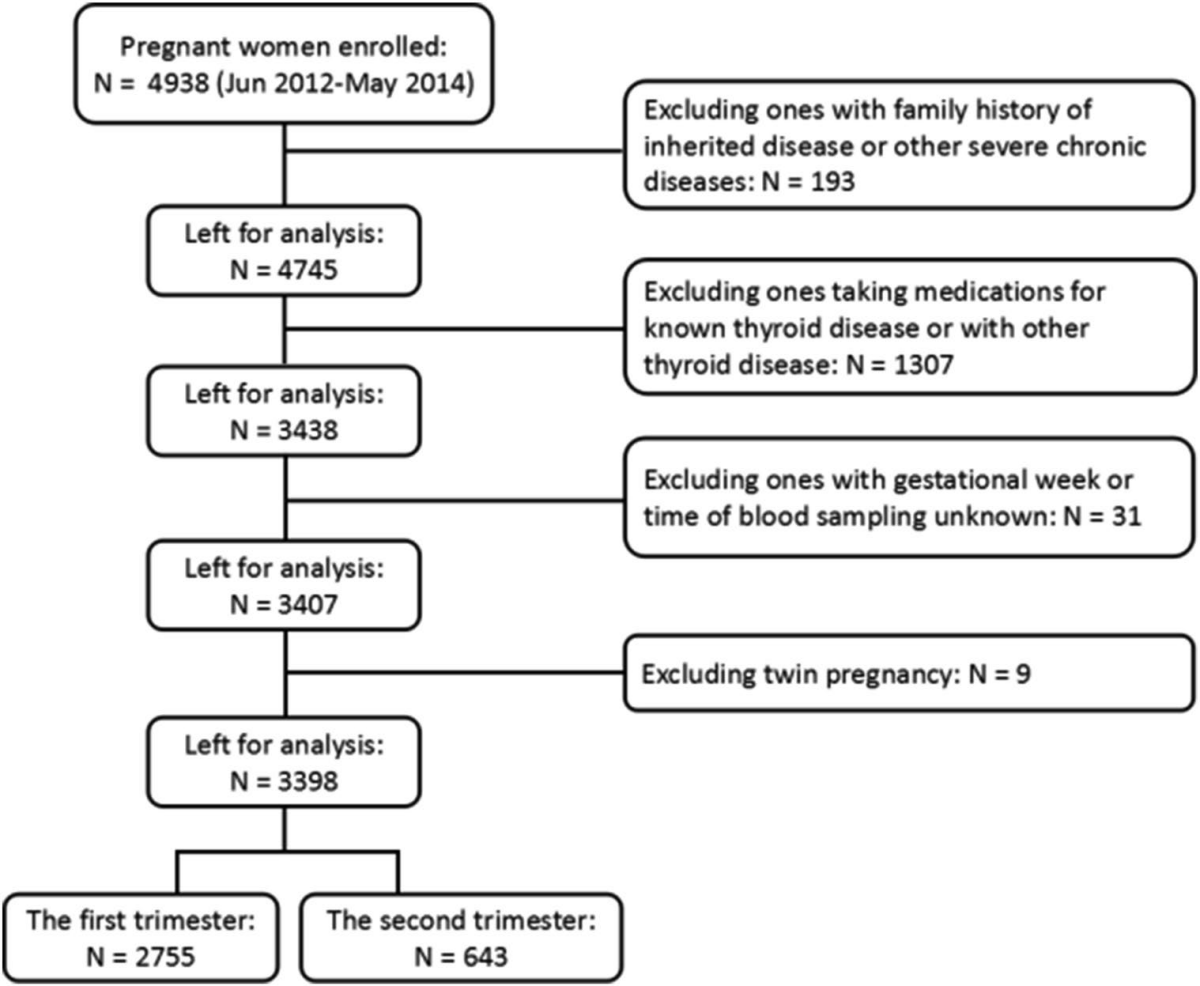
Flowchart of the study population

A total of 3398 pregnant women meeting the criteria were enrolled and divided into five groups on the basis of gestational week and thyroid function. There are three groups in the first trimester including control, IMH with LT4 treatment, and IMH without LT4 treatment. Two groups in the second trimester include IMH and control. The definitions of these groups are provided in the “Diagnostic criteria” section. The flowchart is showed in Fig. 1.

The experimental procedure described here was approved by the Ethics Committee of the China Medical University ([2012] 2011-32-4) and is congruent with the Declaration of Helsinki. Signed informed consent was obtained from every patient who participated.

### Methods

Maternal serum specimens were collected during the first (before 12 weeks’ gestation) and second (12–28 weeks’ gestation) trimester of pregnancy. Serum specimens were obtained in the early morning after an overnight fast. Serum TSH, FT4 and TPOAb levels were measured using electrochemiluminescence immunoassay (Cobas Elesys 601, Roche Diagnostics). The intra-assay coefficients of variation (CV) of TSH, FT4, and TPOAb were 1.57–4.12%, 2.24–6.33%, and 2.42–5.63%, respectively. The inter-assay CVs were 1.26–5.76%, 4.53–8.23%, and 5.23–8.16%, respectively [12].

Participants were fully informed by doctors and were allowed to signal whether to accept L-T4 treatment or not. After being fully informed by a doctor, the choice of whether or not to start levothyroxine treatment was made by the patient. Participants accepting L-T4 intervention began taking 50 μg of levothyroxine daily, the lower oral dose was intended to avoid overtreatment in pregnant women with IMH. The oral dose was adjusted by doctors according to their thyroid function during follow-up, to a maximum of 100 μg daily. The goal was to ensure that TSH and FT4 levels in the participants were within the normal pregnancy-specific reference range.

### Diagnostic criteria

The 2017 Guidelines of the American Thyroid Association recommend that population-based trimester-specific reference ranges should be defined through assessment of local population data. Therefore, we used trimester-specific diagnostic criteria to evaluate the thyroid function of the pregnant women in our study. For the first and second trimesters, the reference intervals for TSH (2.5th–97.5th percentile) were 0.35–4.13 mIU/L and 0.37–4.78 mIU/L, respectively, and the reference intervals for FT4 (2.5th–97.5th percentile) were 13.35–19.01 pmol/L and 10.45–16.22 pmol/L, respectively [12]. A control group was defined as TSH and FT4 within normal range (2.5th–97.5th percentile) and TPOAb < 34 IU/L. IMH was defined as TSH within normal range (2.5th–97.5th percentile) but with FT4 < 2.5th percentile and TPOAb < 34 IU/L. The diagnostic criteria for complications are shown in Supplements.

#### Statistical analysis

Statistical analysis was performed using SPSS version 21.0 (IBM, USA). *p* values < 0.05 were considered statistically significant. Kolmogorov–Smirnov test was used to test normal distribution. Chi-square test was applied to comparison of incidences of adverse pregnancy outcomes. Logistic regression was applied for multifactor analysis. Interactions were evaluated in stratified analyses.

## Results

### Baseline characteristics of subjects

The baseline characteristics of the study population are shown in Tables 1 and 2. A total of 2554 participants had normal thyroid function in the first trimester; 522 had normal thyroid function during both first and second trimesters. Mean age (± SD) at the time of entry was 28.9 ± 3.34 years. The mean birth weight was 3.44 ± 0.46 kg. Women with IMH identified in the first trimester had a higher TSH level (*p* < 0.01) and body mass index (BMI) (*p* < 0.01) compared with control. Women identified with IMH during second trimester tended to be older (*p* = 0.024) and had a higher BMI (*p* < 0.01) in the early pregnancy compared with control.

**Table 1:** Baseline and pregnant outcomes of patients with and without L-T4 intervention and control in first trimester of gestation

	IMH without LT4	IMH with LT4	Control	*p*1	*p*2
*n*	106	95	2554		
Age (years)	28 (27–30)	28 (27–31)	28 (27–31)	0.501	0.837
GW (weeks)	7 (6–7)	6 (6–7)	7 (6–7)	0.178	0.948
BMI (kg/m^2^)	22.9 ± 3.2	23.6 ± 3.4	21.8 ± 3.1	0.172	**< 0.001**
AC (cm)	80.4 ± 8.4	81.7 ± 9.1	78.4 ± 8.6	0.320	0.101
SBP (mmHg)	117 (107–120)	115 (110–124)	116 (110–123)	0.180	0.372
DBP (mmHg)	72 (66–80)	71 (65–80)	71 (66–79)	0.466	0.731
HR (per/min)	80 (74–84)	80 (78–84)	80 (76–84)	0.235	0.085
TSH (mIU/L)	2.29 (1.61–2.98)	2.35 (1.73–3.01)	1.76 (1.22–2.46)	0.397	**< 0.001**
FT4 (pmol/L)	12.29 ± 1.87	12.59 ± 0.54	16.13 ± 1.38	0.846	**< 0.001**
Smoking [*n* (%)]	1 (0.94)	2 (2.11)	71 (2.78)	0.924	0.403
Passive smoking [*n* (%)]	7 (6.60)	17 (17.89)	334 (13.08)	**0.020**	0.051
Alcohol [*n* (%)]	1 (0.94)	8 (8.42)	104 (4.07)	**0.027**	0.172
Multiparous	7 (6.60)	3 (3.16)	110 (4.31)	0.426	0.374
Monthly income (RMB)					
≥ 5000	16 (15.09)	11 (11.58)	368 (14.41)	0.466	0.844
1000–5000	62 (58.49)	79 (83.16)	1834 (71.81)	**< 0.001**	**0.003**
≤ 1000	28 (26.42)	5 (5.26)	352 (13.78)	**< 0.001**	**< 0.001**
Maternal education (college) [*n* (%)]	63 (59.43)	77 (81.05)	1830 (71.65)	**0.001**	**0.007**
Adverse pregnancy outcomes					
Miscarriage	6 (5.66)	12 (12.63)	189 (7.40)	0.084	0.501
Gestational hypertension	5 (4.72)	4 (4.21)	79 (3.09)	1.000	0.514
Eclampsia	1 (0.94)	0 (0)	15 (0.59)	1.000	0.479
GDM	12 (11.32)	19 (20.00)	405 (15.86)	0.070	0.162
Placental abruption	0 (0)	2 (2.11)	6 (0.23)	0.204	1.000
PROM	0 (0)	0 (0)	12 (0.47)	–	1.000
Premature delivery	2 (1.89)	5 (5.26)	83 (3.25)	0.305	0.596
Breech delivery	7 (6.60)	5 (5.26)	108 (4.23)	0.791	0.374
LBW infant	2 (1.89)	4 (4.21)	51 (2.00)	0.516	1.000
Macrosomia	15 (14.15)	8 (8.42)	268 (10.49)	0.276	0.260

Data were expressed as mean ± standard deviation (SD), median (interquartile range [IQR]) or *n* (%). *p*1: IMH without L-T4 vs IMH with L-T4. *p*2: IMH without L-T4 vs Control. *p* values were based on non-parametric test, *t* test, or Chi-square test for categorical variables, *p* < 0.05 was considered as a significant difference, bold fonts indicate significant variables

*GW* gestation week, *BMI* body mass index, *AC* abdomen circumference, *SBP* systolic blood pressure, *DBP* diastolic blood pressure, *HR* heart rate, *TSH* thyroid-stimulating hormone, *FT4* free thyroxine, *GDM* gestational diabetes mellitus, *PROM* premature rupture of fetal membranes, *LBW* low birth weight

**Table 2:** Baseline and pregnant outcomes of patients with IMH and controls in the second trimester of gestation

	IMH	Control	*p*
*n*	121	522	
Age (years)	30 (27–32)	29 (27–31)	**0.024**
GW (weeks)^a^	20 (20–21)	20 (16–20)	**< 0.001**
SBP (mmHg)^a^	113 (106–120)	112 (105–120)	0.876
DBP (mmHg)^a^	70 (63–77)	70 (64–81)	0.329
HR (per/min)^a^	81 (76–92)	80 (74–87)	**0.005**
TSH (mIU/L)^a^	1.73 (1.33–2.28)	1.78 (1.30–2.43)	0.591
FT4 (pmol/L)^a^	9.74 ± 0.58	12.57 ± 1.38	**< 0.001**
GW (weeks)^b^	7 (6–7)	7 (6–7)	0.435
BMI (kg/m^2^)^b^	22.8 ± 3.2	21.9 ± 3.1	**0.003**
AC (cm)^b^	82 (74–89)	78 (72–84)	**0.002**
SBP (mmHg)^b^	114 ± 12.0	113 ± 1.6	0.249
DBP (mmHg)^b^	73 ± 9.8	72 ± 9.1	0.373
HR (per/min)^b^	82 (78–88)	80 (76–87)	**0.028**
TSH (mIU/L)^b^	1.69 (1.13–2.51)	1.61 (1.05–2.33)	0.165
FT4 (pmol/L)^b^	15.21 ± 1.30	16.14 ± 1.35	**< 0.001**
Smoking [*n* (%)]	2 (1.65)	7 (1.34)	1.000
Passive smoking [*n* (%)]	18 (14.88)	54 (10.34)	0.669
Alcohol [*n* (%)]	5 (4.13)	15 (2.87)	0.154
Multiparous	3 (2.48)	14 (2.68)	1.000
Monthly income (RMB)			
≥ 5000	11 (9.09)	61 (11.69)	0.415
1000–5000	79 (65.29)	322 (61.69)	0.461
≤ 1000	31 (25.62)	139 (26.63)	0.821
Maternal education (college) [*n* (%)]	78 (64.46)	327 (62.64)	0.709
Adverse pregnancy outcomes			
Miscarriage	1 (0.83)	4 (0.77)	1.000
Gestational hypertension	10 (8.26)	18 (3.45)	**0.019**
Eclampsia	0 (0)	6 (1.15)	0.509
GDM	23 (19.01)	77 (14.75)	0.268
Placental abruption	0 (0)	2 (0.38)	1.000
PROM	2 (1.65)	3 (0.57)	0.238
Premature delivery	4 (3.31)	17 (3.26)	0.879
Breech delivery	5 (4.13)	17 (3.26)	0.841
LBW infant	2 (1.65)	13 (2.49)	0.830
Macrosomia	26 (21.49)	70 (13.41)	**0.024**

Data were expressed as mean ± standard deviation (SD), median (interquartile range [IQR]) or *n* (%). *p* values were based on non-parametric test, *t* test, or Chi-square test for categorical variables and were compared with control. *p* < 0.05 was considered as a significant difference, bold fonts indicate significant variables

*GW* gestation week, *BMI* body mass index, *AC* abdomen circumference, *SBP* systolic blood pressure, *DBP* diastolic blood pressure, *HR* heart rate, *TSH* thyroid-stimulating hormone, *FT4* free thyroxine, *GDM* gestational diabetes mellitus, *PROM* premature rupture of fetal membranes, *LBW* low birth weight

^a^Index in second trimester

^b^Index in first trimester

### IMH and pregnancy outcomes

Adverse pregnancy outcomes are shown in Tables 1 and 2.

### IMH in first trimester with and without L-T4 treatment

A total of 201 women were identified with IMH in the first trimester. Of these, 95 women chose to accept L-T4 intervention. No statistically differences in adverse pregnancy outcomes were found between women with IMH and controls. The results suggested that L-T4 treatment for IMH before 12 weeks of gestation did not ameliorate adverse pregnancy outcomes. IMH in the first trimester was not associated with increased risk of adverse pregnancy outcomes after corrected by known confounding factor.

### IMH in the second trimester

A total of 121 women had IMH in the second trimester with normal FT4 in the first trimester. Compared with the control group, they had a significantly increased risk of macrosomia (8.26% vs. 3.45%, *p* = 0.022) and gestational hypertension (21.49% vs. 13.41%, *p* = 0.018).

### IMH stratified by BMI

Women with IMH in the second trimester had higher mean BMI in the early pregnancy. A high BMI in the early pregnancy was related to adverse pregnant outcomes; therefore, the participants with IMH in the second trimester were divided into two groups, BMI < 25 kg/m^2^ and BMI ≥ 25 kg/m^2^. Women with IMH with a BMI < 25 kg/m^2^ were more likely to have gestational hypertension (9.78% vs. 2.43%, *p* = 0.002) and macrosomia was more likely (20.65% vs. 11.26%, *p* = 0.013) than for women of BMI < 25 kg/m^2^ without IMH (Table 3).

**Table 3:** Adverse pregnancy outcomes in IHM and controls in the second trimester of gestation according to BMI

	BMI < 25 kg/m^2^		BMI ≥ 25 kg/m^2^	
	IMH	Control	IMH	Control
*n*	92	453	29	69
Miscarriage	1 (1.09)	3 (0.66)	0 (0)	1 (1.45)
Gestational hypertension	9 (9.78)*	11 (2.43)	1 (3.45)	7 (10.14)
Eclampsia	0 (0)	4 (0.88)	0 (0)	2 (2.90)
GDM	18 (19.57)	63 (13.91)	5 (17.24)	14 (20.29)
Placental abruption	0 (0)	2 (0.44)	0 (0)	0 (0)
PROM	2 (2.17)	3 (0.66)	0 (0)	0 (0)
Premature delivery	4 (4.35)	12 (2.65)	0 (0)	5 (7.25)
Breech delivery	5 (5.43)	16 (3.53)	0 (0)	1 (1.45)
LBW infant	2 (2.17)	11 (2.43)	0 (0)	2 (2.90)
Macrosomia	19 (20.65)*	51 (11.26)	7 (24.14)	19 (27.54)

Data given as *n* (%)

*GDM* gestational diabetes mellitus, *PROM* premature rupture of fetal membranes, *LBW* low birth weight

*p < 0.05, *p* values based on Chi-square test for categorical variables and were compared with control

### Logistic regression analysis

Logistic regression analysis indicated IMH as a risk factor for adverse pregnancy outcomes, with BMI in the early pregnancy < 25 kg/m^2^ in women with IMH associated with increased risk of gestational hypertension (aOR 4.203, 95% CI 1.611–10.968, *p* = 0.003; Table 4) and macrosomia (aOR 1.942, 95% CI 1.076–3.503, *p* = 0.027; Table 4). Furthermore, high systolic blood pressure (SBP) in second trimester was related to an increased risk of gestational hypertension (aOR 1.053, 95% CI 1.016–1.092, *p* = 0.005; Table 4) and abdomen circumference (AC) in the early pregnancy was associated with macrosomia (aOR 1.039, 95% CI 1.008–1.070, *p* = 0.012; Table 4).

**Table 4:** Crude and adjusted odds ratio of gestational hypertension and macrosomia in the second trimester of gestation

Outcomes	Unadjusted			Adjusted model 1			Adjusted model 2		
Risk factors	cOR	(95% CI)	*p* value	aOR	(95% CI)	*p* value	aOR	(95% CI)	*p* value
Gestational hypertension									
IMH in second trimester^a^	2.525	1.135–5.621	**0.023**	2.106	0.753–5.893	0.156	2.238	0.885–5.657	0.089
IMH in second trimester (BMI < 25 kg/m^2^)^b^	4.357	1.751–10.842	**0.002**	3.927	1.363–11.314	**0.011**	4.203	1.611–10.968	**0.003**
SBP in second trimester (BMI < 25 kg/m^2^)^b^	1.049	1.013–1.087	**0.008**	1.057	1.008–1.109	**0.022**	1.053	1.016–1.092	**0.005**
Macrosomia									
IMH in second trimester^c^	1.770	1.071–2.925	**0.026**	1.692	0.948–3.020	0.075	1.656	0.943–2.91	0.079
IMH in second trimester (BMI < 25 kg/m^2^)^d^	2.065	1.152–3.700	**0.015**	2.032	1.064–3.880	**0.032**	1.942	1.076–3.503	**0.027**
AC in early pregnancy (BMI < 25 kg/m^2^)^d^	1.041	1.011–1.073	**0.007**	1.044	1.010–1.079	**0.010**	1.039	1.008–1.070	**0.012**

Bold fonts indicate significant variables

*aOR* adjusted odds ratio, *CI* confidence interval, *cOR* crude odds ratio

^a,c^Adjusted model 1: BMI, IMH, smoking, passive smoking, alcohol, GW, AC, SBP, DBP, HR, TSH, maternal education, social-economic status, multiparous

^a^Adjusted model 2: IMH, HR, SBP, BMI; ^c^adjusted model 2: IMH, BMI, HR, gestational week

^b,d^ Adjusted model 1: IMH, smoking, passive smoking, alcohol, GW, AC, SBP, DBP, HR, TSH, maternal education, social-economic status, multiparous

^b^Adjusted model 2: IMH, SBP; ^d^adjusted model 2: AC, IMH

## Discussion

Thyroid dysfunction during pregnancy raises concern about the possibility of adverse outcomes. In this study, we found that IMH identified in the first trimester had no significant effect on pregnancy outcomes, irrespective of whether L-T4 therapy was given. IMH occurring in the second trimester was associated with an increased risk of pregnancy hypertension and macrosomia even when thyroid function was normal during the early pregnancy. The risk persisted after adjusting for potential confounders.

The causes of IMH have not been well understood. The most common one is iodine deficiency [4, 5]. In the present study, all the participants were from iodine-sufficient area. Moreover, China has implemented a salt iodization policy since 1996 to keep the residents remain iodine sufficient. Serum FT4 might be influenced by different assay methods. The classic equilibrium dialysis or ultrafiltration methods, as well as solid-phase extraction–liquid chromatography/tandem mass spectrometry methods have higher reliability and reproducibility [4, 12]. However, these assays are laborious, time-consuming, expensive, and not widely available. Immunoassays are widely used in clinical practice, but it may not exclude possible interference. Our previous study conducted by Zhang et al. [12] has confirmed that the FT4 concentrations measured by immunoassays in our study displayed a consistent tendency with liquid chromatography/tandem mass spectrometry (LC/MS) assays in the study by Kahric et al. [13] and were negatively correlated with TSH (*r* = − 0.201, *p* < 0.01) in the first trimester. Zhang et al. [12] have indicated that the prevalence of hypothyroxinemia in the first and second trimester was 2.0% and 1.2–2.4%, respectively. The current studies showed that the incidence of the IMH was estimated at 2–4.3%, and may up to 25–30% [4]. The incidences of IMH were various due to the different race or ethnicity, iodine nutrition, and diagnostic criteria.

Elevated BMI has been found in pregnancy women with hypothyroxinemia in many studies [14–18]. In our study, participants with IMH identified in the first or second trimester also tended to have a higher BMI recorded in the early pregnancy. Our previous study found that BMI ≥ 25 kg/m^2^ may act as an indicator of hypothyroxinemia [14]. Korevaar et al. suggested that a higher BMI may lead to a lower thyroid functional capacity [15]. To remove the influence of BMI on IMH, our participants were divided into two groups according to their BMI in the early pregnancy and BMI was considered as an effect factor in our analysis.

The previous epidemiological evidence has proved that IMH during the early pregnancy has a negative effect on pregnancy outcomes. The benefits of treating hypothyroxinemia women in terms of obstetric complications have not been investigated. Casey et al. [16] conducted a randomized, placebo-controlled trial involved 526 cases of women with a singleton pregnancy before 20 weeks of gestation for hypothyroxinemia and found that mothers with hypothyroxinemia on L-T4 treatment did not have a better pregnancy and neonatal outcomes than controls, which is consistent with our results. However, thyroid hormone replacement therapy in their study was initiated at a median gestational age of 18 weeks; the mean gestational age for treatment initiation in our study was 6.27 weeks. However, our study could not exclude the possibility that there was confounding by indication.

We concluded that women with normal thyroid function in the early pregnancy but with IMH in the second trimester had an increased risk of gestational hypertension. IMH was a risk factor for gestational hypertension when BMI in the early pregnancy was < 25 kg/m^2^. Medici et al. [19] showed in their study that, within the normal range, high–normal FT4 levels were associated with an increased risk of gestational hypertension (OR 1.62, 95% CI 1.06–2.47), but they did not find any relationship between hypothyroxinemia in the early pregnancy and gestational hypertension. However, they were unable to fully correct for factors known to be associated with thyroid parameters. A prospective population-based cohort Northern Finland Birth Cohort 1986 (NFBC 1986) found that hypothyroxinemia before 20 gestational weeks was not at increased risk of gestational hypertension [20]. Another large-population study indicated that hypothyroidism but not hypothyroxinemia was associated with hypertensive disorders [21].

Our results suggested that women with IMH during the second trimester had an increased incidence of macrosomia (aOR 1.942, 95% CI 1.076–3.503, *p* = 0.027) when the early pregnancy BMI was < 25 kg/m^2^. In 2007, Casey et al. [8] analyzed 233 women and found no excessive adverse pregnancy outcomes in women who had IMH in the first half of pregnancy. Despite this negative result, which may have been due to differences in women’s average age and ethnicity, most studies have clearly indicated adverse pregnancy outcomes for IMH. European research that included 879 pregnant women found that IMH during the early pregnancy was associated with an increased risk of macrosomia compared with a control group [17]. Women with hypothyroxinemia had significantly increased BMI from preconception to the time of delivery. Isolated hypothyroxinemia was associated with an increased maternal BMI. Their logistic regression analysis suggested that BMI was not a risk factor for macrosomia. In that study, IMH was defined as a normal maternal TSH concentration and an FT4 concentration in the lower 2.5th–5th percentile of the pregnancy reference range. Goldman et al. [22] found that maternal hypothyroxinemia during the early pregnancy increased the risk of macrosomia (aOR 1.97; 95% CI 1.37–2.83) after adjusting for maternal age, prior pregnancy, BMI, and study site. Incidence of pregnancy hypertension of women with hypothyroxinemia in the second trimester was increased compared with controls (9.5% vs. 5.4%), although not significantly. One population-based birth cohort study, embedded in the Generation R study, assessed the thyroid status of 4894 women in the early pregnancy (median of gestation week was 13.4) and concluded that maternal hypothyroxinemia was associated with higher fetal birth weight [23]. However, the women with hypothyroxinemia were older than the controls, as well as less educated and more likely to smoke during their pregnancy. The Ma’anshan Birth Cohort (MABC) Study in China demonstrated that the risk of having a baby large for gestational age was increased among women with IMH in the second trimester (OR 2.088, 95% CI 1.193–3.654) [18]. A study in Henan, China found that women with IMH had a significant increase odd of having macrosomia (aOR 2.22, 95% CI 1.13–4.85) [24]. Maternal obesity may play a mediating effect between IMH and macrosomia. However, one limitation of that study is that the collected samples were from different gestational age. However, Hamm et al. [25] in Canada observed that IMH was not related to any adverse effects on fetal growth or pregnancy outcome.

Our study is one of only a few focused on associations between pregnancy outcomes and IMH diagnosed with trimester-specific diagnostic criteria in the first and second trimesters. In addition, we compared pregnancy outcomes in women with IMH in first trimester who were assigned L-T4 treatment before 12 gestational weeks and those who were not.

Our study was limited that some of the events including placental abruption, PROM, and eclampsia were infrequent. No participants were performed assessment of the T3 level. Another limitation was that the treatment in our study was not randomized. The results may be affecting by confounding factors, although some factors had been corrected. Besides, iodine concentrations were absent in our study. In addition, the study only focused on the first and second trimester of pregnancy.

## Conclusions

We conclude that IMH identified in the second trimester of pregnancy is associated with an increased risk of adverse pregnancy outcomes. IMH with L-T4 treatment during the early pregnancy did not appear to improve outcomes, but residual confounding cannot be excluded. The follow-up of pregnant women’s thyroid function during the second trimester of pregnancy appears to be important, even if the early pregnancy thyroid function is normal. Although thyroid dysfunction of the second trimester evidently increases the risk of adverse pregnancy outcomes, further research into whether intervention at this time can reverse adverse outcomes is required, to provide evidence for use in clinical management.

## Electronic supplementary material

Below is the link to the electronic supplementary material.

Supplementary material 1 (DOCX 12 kb)
