# Supplementary material for: The impact of isolated maternal hypothyroxinemia during the first and second trimester of gestation on pregnancy outcomes: an intervention and prospective cohort study in China
Source: J Endocrinol Invest. 2018 Oct 17;42(5):599–607. doi: 10.1007/s40618-018-0960-7 (PMC6476837; doi:10.1007/s40618-018-0960-7)
Supplement: Supplementary file 1 — Supplementary material 1 (DOCX 12 kb) [file 40618_2018_960_MOESM1_ESM.docx]

| **Complications and diagnostic criteria** | | | |
| --- | --- | --- | --- |
| *Complications* |  | *Diagnostic criteria* |  |
| *Gestational hypertension* | BP>140/90 mmHg, urine protein (-) | | |
| *Eclampsia* | BP>140/90 mmHg, urine protein (+) | | |
| *GDM* | 75g OGTT, FBG>5.1 mmol/L; 1h PG>10.0 mmol/L; 2h PG>8.5 mmol/L; any of the above reaching the diagnostic criteria can be diagnosed GDM | | |
| *Miscarriage* | Pregnancy loss, spontaneous abortion or stillbirth before 28 weeks of gestation | | |
| *Placental abruption* | Separation of a normally implanted placenta prior to or during delivery | | |
| *PROM* | Rupture that occurs prior to labor, regardless of the gestational age | | |
| *Premature delivery* | Delivery before 37 weeks of gestation | | |
| *Breech delivery* | According to obstetrician | | |
| *LBW infant* | Birth weight <2500 g | | |
| *Macrosomia* | Birth weight >4000 g | | |
| BP, blood pressure; OGTT, oral glucose tolerance test; FBG, fasting blood glucose; PG, postprandial glucose; GDM, gestational diabetes mellitus;  LBW, low birth weight; PROM, premature rupture of fetal membranes. | | | |
